# Metamizole relieves pain by influencing cytokine levels in dorsal root ganglia in a rat model of neuropathic pain

**DOI:** 10.1007/s43440-020-00137-8

**Published:** 2020-07-20

**Authors:** Renata Zajaczkowska, Klaudia Kwiatkowski, Katarzyna Pawlik, Anna Piotrowska, Ewelina Rojewska, Wioletta Makuch, Jerzy Wordliczek, Joanna Mika

**Affiliations:** 1 Department of Interdisciplinary Intensive Care, Jagiellonian University Medical College, Kraków, Poland; 2 Department of Pain Pharmacology, Maj Institute of Pharmacology, Polish Academy of Sciences, 12 Smetna Str., 31-343 Kraków, Poland

**Keywords:** Interleukins, Chemokines, IL-1beta, IL-18, IL-6, CCL2, CCL4, CCL7, Metamizole

## Abstract

**Background:**

Treatment of neuropathic pain is still challenging. Recent studies have suggested that dorsal root ganglia (DRG), which carry sensory neural signals from the peripheral nervous system to the central nervous system, are important for pathological nociception. A proper understanding of the significance and function of DRG and their role in pharmacotherapy can help to improve the treatment of neuropathic pain. Metamizole, also known as sulpyrine or dipyrone, is a non-opioid analgesic commonly used in clinical practice, but it is not used for neuropathic pain treatment.

**Methods:**

Chronic constriction injury (CCI) of the sciatic nerve was induced in Wistar rats. Metamizole was administered intraperitoneally (ip) preemptively at 16 and 1 h before CCI and then twice a day for 7 days. To evaluate tactile and thermal hypersensitivity, von Frey and cold plate tests were conducted, respectively.

**Results:**

Our behavioral results provide evidence that repeated intraperitoneal administration of metamizole diminishes the development of neuropathic pain symptoms in rats. Simultaneously, our findings provide evidence that metamizole diminishes the expression of pronociceptive interleukins (*IL*-*1beta*, *IL*-*6*, and *IL*-*18*) and chemokines (*CCL2*, *CCL4*, and *CCL7*) in DRG measured 7 days after sciatic nerve injury. These assays indicate, for the first time, that metamizole exerts antinociceptive effects on nerve injury-induced neuropathic pain at the DRG level.

**Conclusions:**

Finally, we indicate that metamizole-induced analgesia in neuropathy is associated with silencing of a broad spectrum of cytokines in DRG. Our results also suggest that metamizole is likely to be an effective medication for neuropathic pain.

**Graphic abstract:**

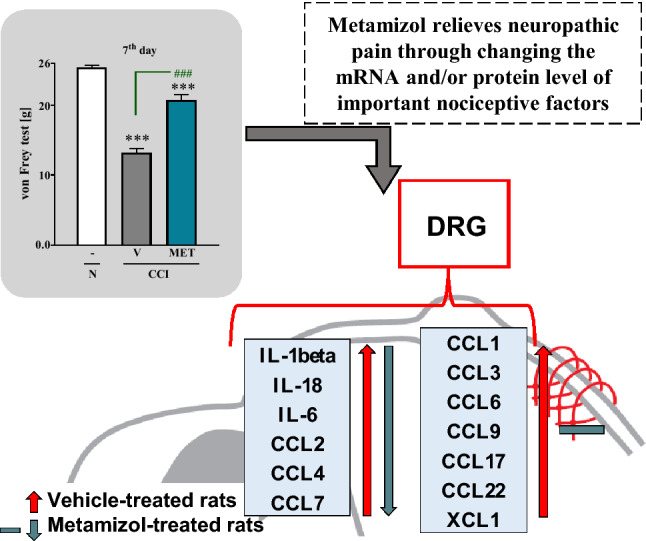

## Introduction

Chronic pain occurs in approximately 20% of the general population [1], and the prevalence of neuropathic pain is 6.9% [2]. In a review [3] based on 174 published clinical trials, it was reported that there are no drug treatments available that can relieve all neuropathic pain conditions. Recently, accumulating evidence has demonstrated that neuroinflammation at the level of dorsal root ganglia (DRG) is strongly involved in peripheral nerve injury-induced pain [4–11]. DRG contain the greatest proportion of sensory neurons, the cells that are primarily responsible for transduction of sensory information from the periphery and for transmitting this information to the central nervous system. DRG are active participants in peripheral processes, including inflammation and neuropathic pain development. Current methods for reducing neuropathic pain directly in DRG include ablation via continuous thermal radiofrequency, pulsed radiofrequency, or electrical neurostimulator technologies and modification of DRG cellular function using viral vectors and gene silencing [12]. Pharmacological blockade of DRG via intraforaminal epidural administration of lidocaine results in relief of painful sensations in patients with phantom limb pain [13]. Therefore, DRG neurons seem to be an ideal target for treatment of chronic pain, and it is important to study the role of neuroimmune interactions in DRG after the administration of analgesics. Nerve injury activates nociceptive pathways and changes gene expression in DRG neurons, which may contribute to the development and maintenance of neuropathic pain. Current studies have described immune-related factors in DRG that are key players in the sensitization occurring under neuropathic pain [7–11, 14].

Metamizole (dipyrone, sulpyrine) is a non-opioid analgesic with antispasmodic and antipyretic properties. Metamizole was introduced in the clinic for the first time in 1922 in Germany and has since been used worldwide for treatment of several painful conditions, including postsurgical pain, colic pain and migraine [15–17]. Although metamizole has been used in the clinic for many years, its mechanisms of action are not entirely clear or fully understood. This drug is used to relieve somatic and visceral pain but not to treat neuropathic pain, although some of its mechanisms of action indicate potential therapeutic properties. The reason for this state of affairs is primarily the small number of basic experimental studies and the even smaller number of clinical trials assessing the efficacy of metamizole in the treatment of neuropathic pain, and therefore, there is a lack of supporting evidence for placing metamizole in the group of drugs recommended for neuropathy therapy. Until now, it was suggested that the molecular mechanisms underlying the effects of metamizole involve the endogenous opioid [18] and endocannabinoid [19] systems, the periaqueductal gray matter–rostral ventromedial medulla axis [20], and inhibition of COX-1/COX-2 [21]. Recently, it has been shown that metamizole in the brain reduces microglial activation and, thus, has a neuroprotective effect in a model of experimental ischemia [22]. Our previous results provide evidence that repeated intraperitoneal administration of metamizole for 7 days diminished neuropathic pain-related symptoms in mice after sciatic nerve injury. Moreover, we showed that its effect is associated with silencing of spinal macrophage/microglial activation, consequently reducing the levels of pronociceptive cytokines (IL-1beta, XCL1, and CCL2) [23]. Further, metamizole exerts very good analgesic effects after intraperitoneal administration, and thus, we hypothesized that its effectiveness is likely due to changes not only at the level of the spinal cord but also in DRG.

Therefore, the goal of our current work was to investigate the influence of preemptive and repeated (for 7 days) intraperitoneal injection of metamizole on the development of tactile and thermal hypersensitivity in rats and its influence on cytokine changes at the level of DRG after chronic constriction injury (CCI) of the sciatic nerve (Bennett model) [24]. The purpose of the research was to demonstrate for the first time using RT-qPCR and/or Western blot analysis how metamizole administration influences the levels of important pronociceptive interleukins (*IL*-*1beta*, *IL*-*6*, and *IL*-*18*) and chemokines (*CCL1*, *CCL2*, *CCL3*, *CCL4*, *CCL6*, *CCL7*, *CCL9*, *CCL17*, and *CCL22*) in DRG 7 days after CCI.

## Materials and methods

### Animals and ethical statement

Male Wistar rats (270–300 g) from Charles River Laboratories International, Inc. (Germany) were employed for the present study. The rats were housed in cages lined with sawdust under a standard 12-/12-h light/dark cycle (lights on at 8.00 a.m.) with food and water available ad libitum. The animals were allowed to adapt to the environment for approximately 5 min prior to behavioral testing. All experiments were conducted according to the guidelines of the International Association for the Study of Pain (IASP) [25], our previous studies [8–10, 26] and the National Institutes of Health (NIH) Guide for the Care and Use of Laboratory Animals. The experimental protocol was duly approved by the II Local Bioethics Committee branch of the National Ethics Committee for Experiments on Animals-based at the Maj Institute of Pharmacology, Polish Academy of Sciences (Krakow, Poland), permission numbers 1277/2015 and 153/2016. Care was taken to minimize animal suffering and limit the number of animals used in the experiments (3R policy).

### Chronic constriction injury of the sciatic nerve

Peripheral neuropathic pain was induced in rats by CCI. In brief, CCI was performed under sodium pentobarbital anesthesia (60 mg/kg; ip) according to the method described in [24] and in our previous publications [8–10, 26]. An incision was made below the right hip bone, and subsequently, the right sciatic nerve was exposed by separation of the *biceps femoris* and *gluteus superficialis*. To produce the injury, four ligatures were made by loosely tying 4/0 silk thread around the sciatic nerve with 1-mm spacing until a brief twitch in the respective hind limb was observed. After the nerve ligation, all rats developed long-lasting neuropathic pain.

### Drug administration

Metamizole (**Met**, 500 mg/kg; SANOFI, Germany) was dissolved in water for injection and administered intraperitoneally (ip) preemptively 16 and 1 h before CCI and then twice a day for 7 days. The dose of metamizole was chosen based on our previous research [23] and the available literature [27, 28]. The control group received vehicle (water for injection) according to the same schedule. Before drug injections, the baseline behaviors of the animals were determined using von Frey and cold plate tests. No adverse side effects of metamizole treatment were observed during the experiments.

### Behavioral tests

Pain hypersensitivity tests were conducted 60 min after the final morning ip injection of metamizole 2 and 7 days after CCI, always in the same order: the von Frey test followed by the cold plate test.

#### Tactile sensitivity (von Frey test)

Hypersensitivity to mechanical stimuli was measured in naive rats and in the rats subjected to CCI using an automated von Frey apparatus (Dynamic Plantar Anesthesiometer, cat. no. 37400, Ugo Basile, Italy) as previously described [9, 10, 14]. Five minutes before the experiment, the animals were placed in transparent plastic cages with wire net floors to allow for behavioral accommodation. A series of von Frey stimuli up to 26 g were used in our experiments. The mid-plantar area of the ipsilateral and contralateral hind paws was tested, and measurements were recorded automatically as described previously [29]. No significant differences in contralateral hind paw responses were observed between the rats subjected to CCI and the naive rats. Tactile hypersensitivity was assessed 60 min after the final drug administration. The ipsilateral paw was tested twice at 2-min intervals, and the mean value was calculated. No significant differences in paw reactions were observed between the contralateral hind paws of rats with CCI and naive rats.

#### Thermal sensitivity (cold plate test)

Hypersensitivity to thermal stimuli was measured using a cold plate apparatus (Cold/Hot Plate Analgesia Meter, cat. no. 05044/230 VAC, Columbus Instruments, USA) as described previously [29]. The temperature of the cold plate was kept at 5 °C, and the cut-off latency was set at 30 s. For the experiments, the animals were placed on the cold plate, and the time to lift the hind paw was recorded.

### Gene expression analysis using RT-qPCR

For quantitative real-time PCR (RT-qPCR) analysis, tissue derived from DRG (L4–L6, pooled into one sample) was collected from naive rats and CCI rats immediately after decapitation on day 7 after repeated metamizole or vehicle treatment. The collected tissue was placed individually in tubes containing RNAlater (Qiagen Inc.) and stored at − 80 °C until isolation of RNA. Total RNA extraction was performed using TRIzol (Invitrogen, Germany) based on the protocol described by Chomczynski and Sacchi [30]. The RNA quality and concentration were measured with a DeNovix DS-11 spectrophotometer (DeNovix Inc., Wilmington, USA). An Omniscript RT Kit (Qiagen Inc., Hilden, Germany) was used for reverse transcription, which was performed at 37 °C for 60 min using 1 μg of total RNA. The reverse transcription reaction mix contained RNAse inhibitor (rRNasin, Promega, Mannheim, Germany) and oligo (dT16) primer (Qiagen Inc., Hilden, Germany). The resulting cDNA was diluted 1:10 with RNase-/DNase-free H_2_O, and ~ 50 ng of cDNA from each sample was used for each RT-qPCR reaction. RT-qPCR was conducted using Assay-On-Demand TaqMan probes based on the manufacturer’s protocol (Applied Biosystems, Foster City, CA, USA) and run in an iCycler device (Bio-Rad, Hercules, Warsaw, Poland). The following TaqMan primers and probes were used: Rn01527840_m1 (*Hprt*); Rn00570480_m1 (*C1q*); Mm01253033_m1 (*GFAP*); Rn00580432_m1 (*Il1b*); Rn01422083_m1 (*Il18*); Rn00561420_m1 (*Il6*); Rn01752376_m1 (*Ccl1*); Rn00580555_m1 (*Ccl2*); Rn01464736_g1 (*Ccl3*); Rn00671924_m1 (*Ccl4*); Rn01456400_m1 (*Ccl6*); Rn01467286_m1 (*Ccl7*); Rn01471276_m1 (*Ccl9*); Rn01536936_g1 (*Ccl17*); Rn01536591_m1 (*Ccl22*); Rn00592605_m1 (*Xcl1*). To determine the amplification efficiency for each assay (between 1.7 and 2), a standard dilution curve was used. The cycle threshold values were calculated automatically using CFX Manager v.2.1 software based on the default parameters. RNA content was calculated as 2^−(threshold cycle)^. The *Hprt* gene was used as an endogenous control, and its expression did not significantly change across the groups (data not shown).

### Western blot analysis of protein levels

Ipsilateral DRG fragments (L4–L6, pooled into one sample) were collected after decapitation, 6 h after the last administration of metamizole on the 7th day post-CCI. First, the tissue lysates were placed in RIPA buffer supplemented with a protease inhibitor cocktail and then cleared via centrifugation (14,000×*g* for 30 min at 4 °C). All samples (20 μg of protein) were heated in loading buffer (4× Laemmli Buffer, Bio-Rad, Warsaw, Poland) for 8 min at 98 °C. Next, the samples were resolved on 4–15% Criterion™ TGX™ precast polyacrylamide gels (Bio-Rad, Warsaw, Poland) and transferred to Immune-Blot PVDF membranes (Bio-Rad, Warsaw, Poland) using a semidry transfer system (30 min, 25 V). The membranes were blocked with 5% nonfat dry milk (Bio-Rad, Warsaw, Poland) in Tris-buffered saline with 0.1% Tween 20 (TBST) for 1 h at room temperature, washed with TBST, and incubated overnight at 4 °C with the following commercially available primary antibodies: anti-Iba-1 (1:1000, Proteintech 10904-1-AP), anti-GFAP (1:10,000, Novus NB300-141), anti-IL-1beta (1:500, Abcam ab9787), anti-IL-18 (1:500, Abcam ab191860), and anti-IL-6 (1:500, Proteintech 21685-1AP). Then, the membranes were incubated with 1:5000 dilutions of horseradish peroxidase-conjugated anti-rabbit secondary antibody for 1 h. The solutions from a SignalBoost™ Immunoreaction Enhancer Kit (Merck Millipore Darmstadt, Germany) were applied to dilute the used antibodies. Afterwards, the membranes were washed using TBST. The immune complexes were detected using Clarity™ Western ECL Substrate (Bio-Rad, Warsaw, Poland) and visualized using a Fujifilm LAS-4000 FluorImager system. Fujifilm Multi Gauge software was used to estimate the density of immunoreactive bands.

### Statistical analysis

**Fig. 1:**
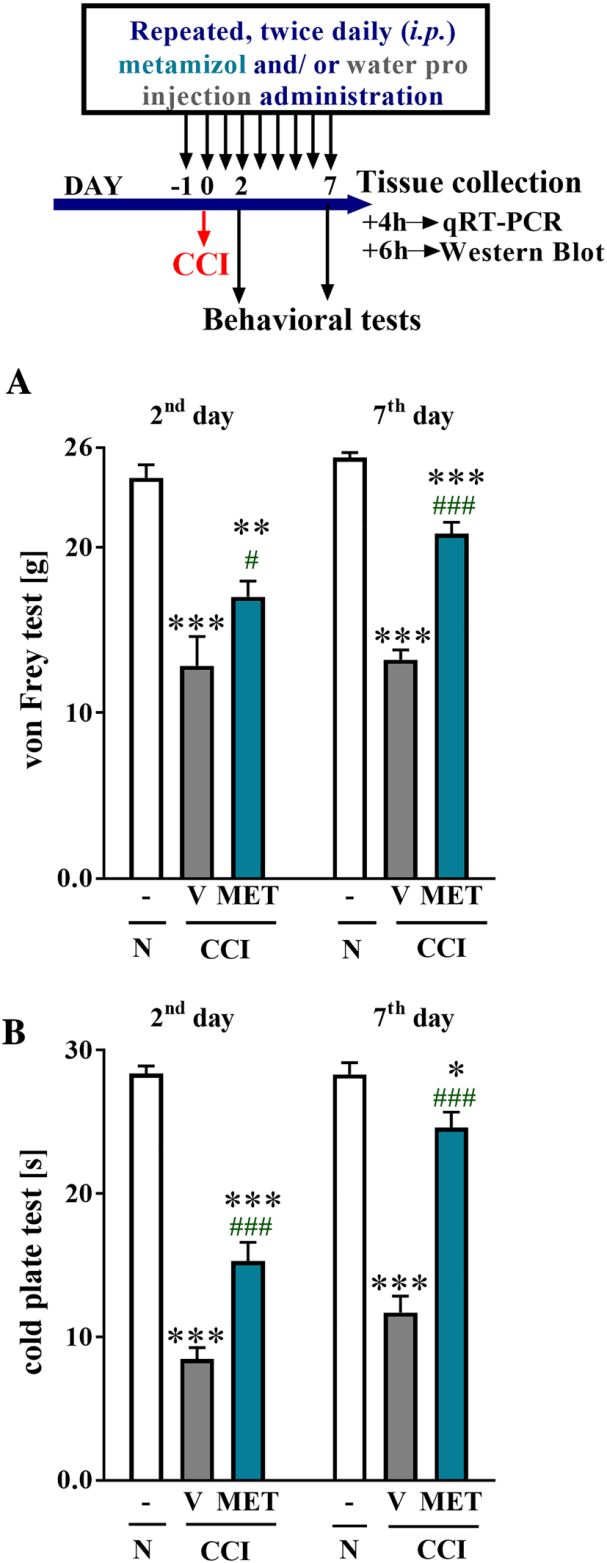
The effects of metamizole administration on neuropathic pain symptoms in rats after CCI. Metamizole (500 mg/kg, ip) was injected 16 h and 1 h before CCI and then twice a day for 7 days. Behavioral evaluations were performed 1 h after the final morning drug administration on days 2 and 7 after CCI. The influence of repeated metamizole administration on the development of mechanical (**a** von Frey test) and thermal (**b** cold plate test) hypersensitivity in rats exposed to CCI was evaluated. The data are presented as the mean ± SEM. On day 2, *n* = 10 rats per group were evaluated. On day 7, the numbers of rats evaluated per group were as follows: *n* = 10 in the naive group; *n* = 19–20 in the VEH- and MET-treated groups. Intergroup differences were analyzed using one-way ANOVA followed by Bonferroni’s multiple comparison test. *p < 0.05, **p < 0.01, and ***p < 0.001 indicate significant differences compared with the naive group; ^#^*p* < 0.05 and ^###^*p* < 0.001 indicate significant differences compared with the vehicle-treated rats after CCI. Additionally, the results were evaluated using one-way ANOVA with repeated measurements to determine differences in behavioral effects between examined time points and using two-way ANOVA to determine the time × drug interaction. *CCI* chronic constriction injury, *MET* metamizole, *VEH* vehicle, *N* naive

**Fig. 2:**
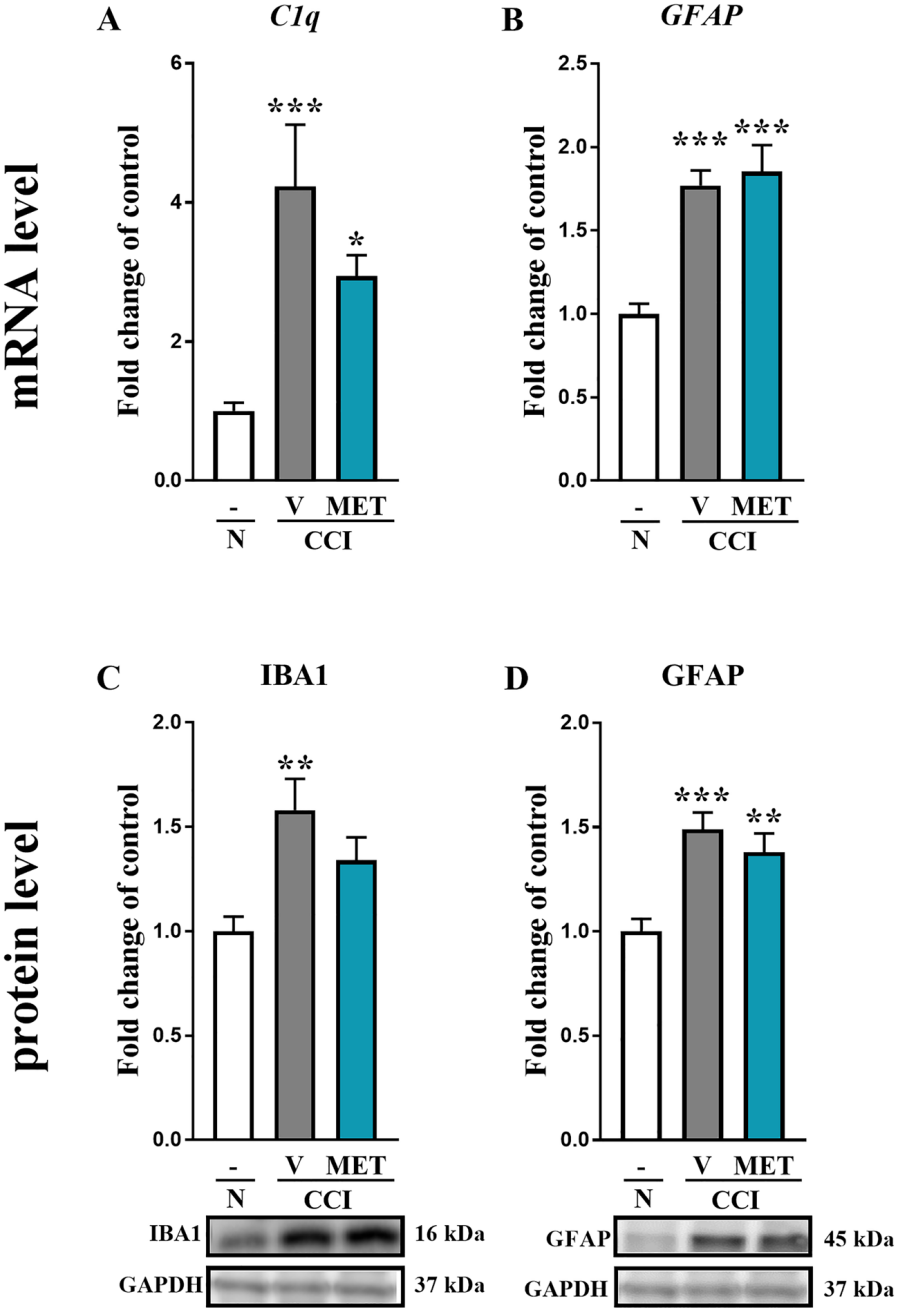
The effects of metamizole administration on the mRNA and protein levels of C1q/IBA1 and GFAP in the dorsal root ganglia (DRG) of rats after CCI. The effects of repeated metamizole administration (500 mg/kg, ip, 16 and 1 h before CCI and then twice a day for 7 days) on the mRNA (upper panel) and protein (lower panel) levels of the glial markers C1q (**a**), IBA-1 (**c)**, and GFAP (**b**, **d**) in DRG 7 days after CCI. The data are presented as the mean ± SEM of 6–8 samples from each group. Intergroup differences were analyzed using one-way ANOVA with Bonferroni’s multiple comparison test. *p < 0.05, **p < 0.01 and ***p < 0.001 indicate significant differences compared with naive rats. *CCI* chronic constriction injury, *MET* metamizole, *VEH* vehicle, *N* naive

**Fig. 3:**
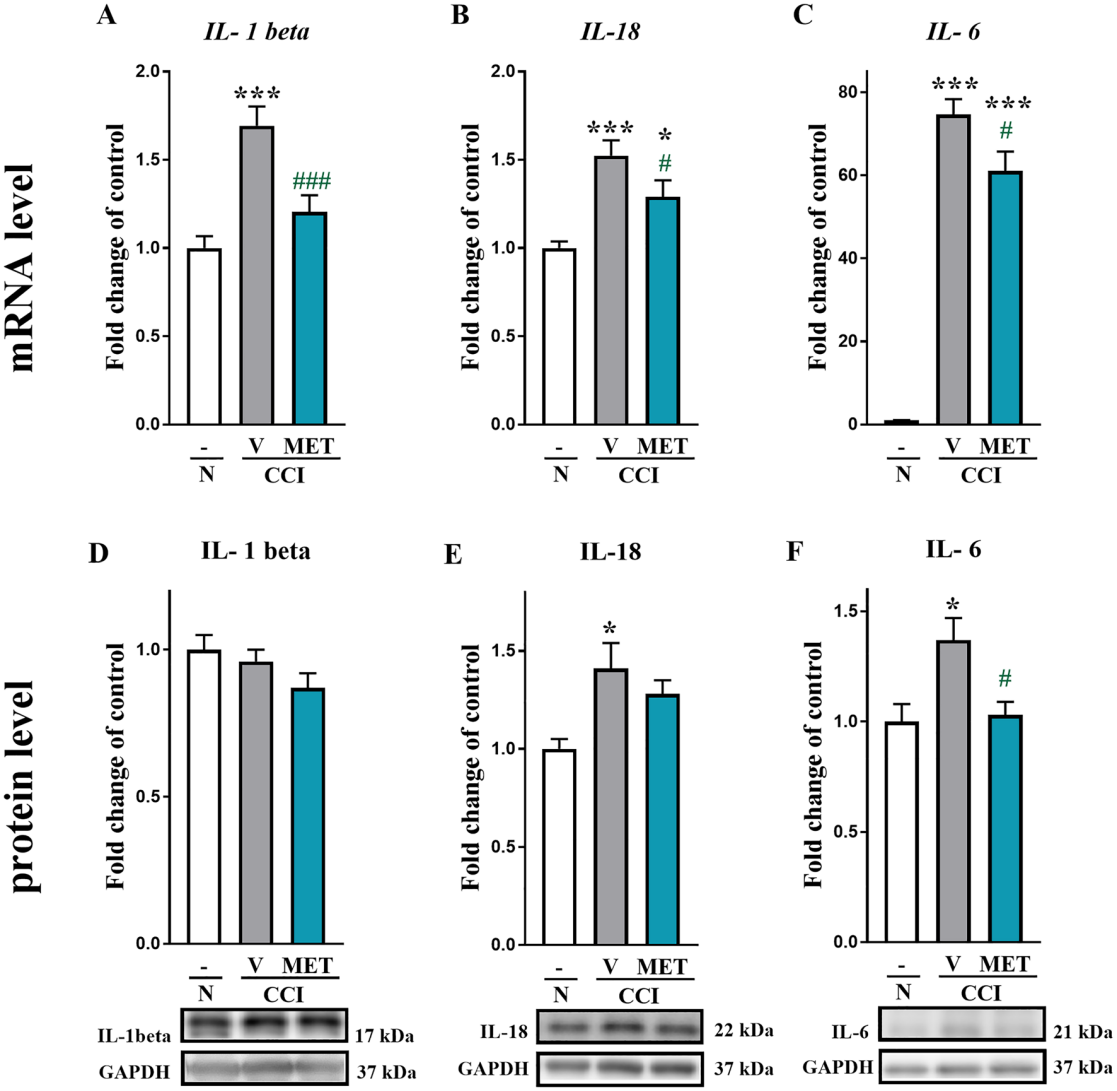
The effects of metamizole administration on the mRNA and protein levels of pronociceptive factors in the dorsal root ganglia (DRG) of rats after CCI. The effects of repeated metamizole administration (500 mg/kg, ip, 16 and 1 h before CCI and then twice a day for 7 days) on the mRNA (upper panel) and protein (lower panel) levels of the pronociceptive factors IL-1beta (**a**, **d**), IL-18 (**b**, **e**), and IL-6 (**c**, **f**) in DRG 7 days after CCI. The data are presented as the mean ± SEM of 6–8 samples from each group. Intergroup differences were analyzed using one-way ANOVA with Bonferroni’s multiple comparison test. *p < 0.05 and ***p < 0.001 indicate differences compared with naive rats. ^#^*p* < 0.05 and ^###^*p* < 0.001 indicate differences compared with vehicle-treated rats after CCI. *CCI* chronic constriction injury, *MET* metamizole, *VEH* vehicle, *N* naive

**Fig. 4:**
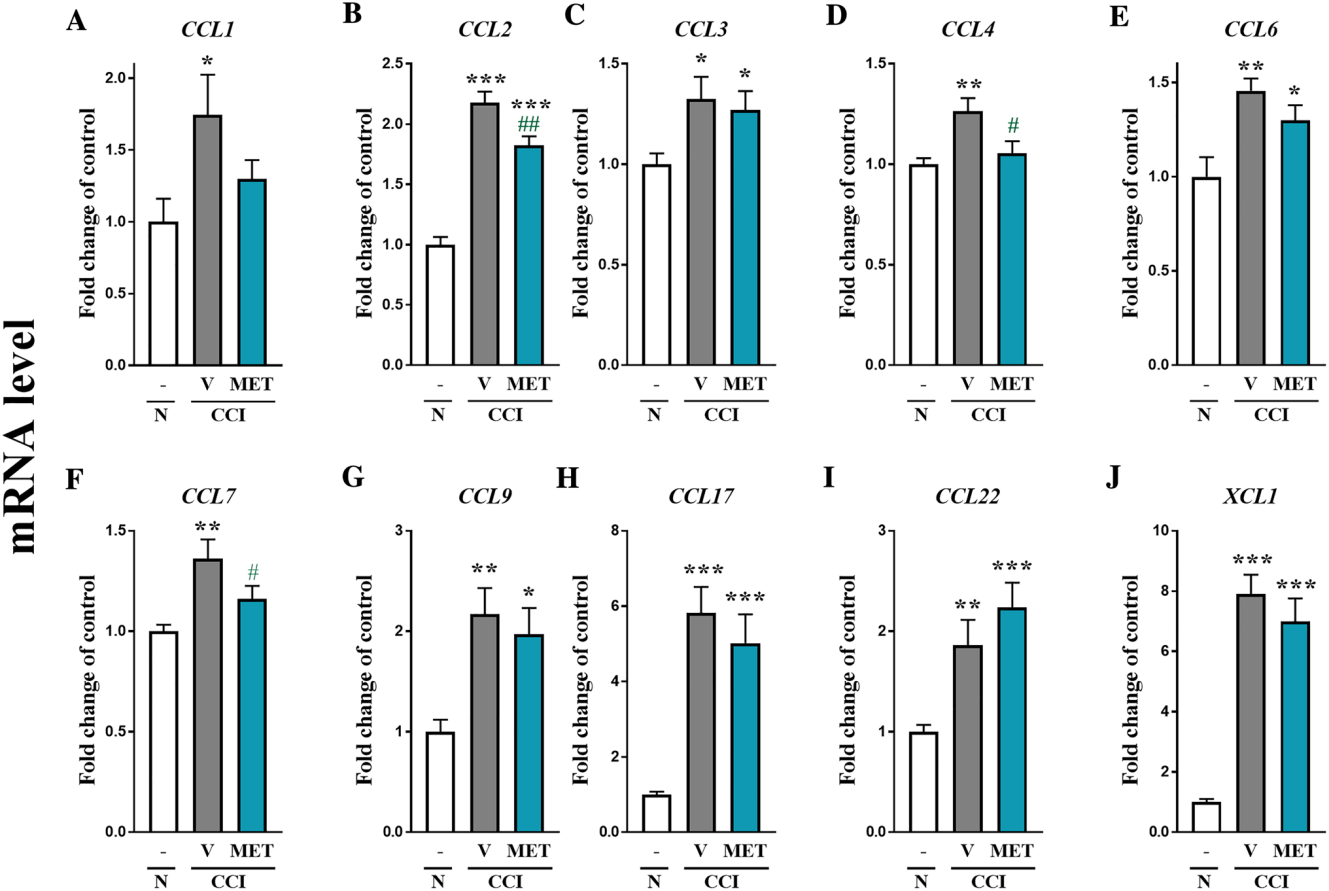
The effects of metamizole administration on mRNA levels of chemokines in the dorsal root ganglia (DRG) of rats after CCI. The effects of repeated metamizole administration (500 mg/kg, ip, 16 h and 1 h before CCI and then twice a day for 7 days) on the mRNA levels of *CCL1* (**a**), *CCL2* (**b**), *CCL3* (**c**), *CCL4* (**d**), *CCL6* (**e**), *CCL7* (**f**), *CCL9* (**g**), *CCL17* (**h**), *CCL22* (**i**), and *XCL1* (**j**) in DRG 7 days after CCI. The data are presented as the mean ± SEM of 6–8 samples from each group. Intergroup differences were analyzed using one-way ANOVA with Bonferroni’s multiple comparison test. *p < 0.05, **p < 0.01 and ***p < 0.001 indicate differences compared with naive rats. ^#^*p* < 0.05 and ^##^*p* < 0.01 indicate differences compared with vehicle-treated rats after CCI. *CCI* chronic constriction injury, *MET* metamizole, *VEH* vehicle, *N* naive

The behavioral data (Fig. 1) are presented as the mean ± SEM. Sample sizes were determined based on our previous experiments. The results were evaluated using one-way ANOVA followed by Bonferroni’s multiple comparisons post hoc test of selected pairs. Additionally, the results were evaluated using one-way ANOVA with repeated measurements to assess differences in behavioral effects between two measured time points and using two-way ANOVA to determine the time × drug interaction. The RT-qPCR/Western blot analysis data (Figs. 2, 3, 4) are presented as the fold change relative to the controls (naive rats) ± SEM and were calculated for the ipsilateral side of the DRG. Differences between the groups were evaluated using one-way ANOVA followed by Bonferroni’s multiple comparisons post hoc test. All graphs were constructed and statistical analyses were performed using Prism software (GraphPad Software, Inc., USA). Differences between the group means were considered significant when *p* < 0.05.

## Results

### The influence of metamizole on pain-related behaviors 2 and 7 days after CCI

The effects of repeated ip administration of metamizole (500 mg/kg) were studied in rats after CCI (Fig. 1a, b). Naive animals reacted to mechanical stimuli weighing 24.2 ± 0.8 g (on the 2nd day) and 25.4 ± 0.3 g (on the 7th day) as measured by the von Frey test and responded to low temperature after 29 ± 0.5 s of exposure (on the 2nd day) and 28.3 ± 0.8 s of exposure (on the 7th day) in the cold plate test. Two and seven days after CCI, all rats exhibited strong mechanical (12.8 ± 1.7 and 13.2 ± 0.6 g, respectively; Fig. 1a) and potent thermal (8.4 ± 0.8 and 11.7 ± 1.1 s, respectively; Fig. 1b) hypersensitivity in the paw ipsilateral to the injury. Metamizole significantly diminished neuropathic pain symptoms on day 2 and even more strongly on day 7 after CCI compared with vehicle treatment as measured by the von Frey (17 ± 0.9 g vs. 12.8 ± 1.7; *F*_(2,27)_ = 22.5; *p* < 0.0001 and 20.8 ± 0.7 g vs. 13.2 ± 0.6; *F*_(2,46)_ = 83.7; *p* < 0.0001, respectively; Fig. 1a) and cold plate (15.3 ± 1.3 s vs. 8.4 ± 0.7; *F*_(2,29)_ = 97.6; *p* < 0.0001 and 24.6 ± 1.1 s vs. 11.7 ± 1.1; *F*_(2,46)_ = 59.5; *p* < 0.0001, respectively; Fig. 1b) tests. One-way ANOVA with repeated measurements revealed that there was no significant difference in the level of hypersensitivity between the 2nd and 7th days after CCI in both the von Frey (*F*_(1,2)_ = 3.044, *p* = 0.2232) and cold plate (*F*_(1,2)_ = 2.303, *p* = 0.2684) tests. However, importantly, two-way ANOVA confirmed a significant interaction between the investigated treatment and the investigated time points only in the cold plate test (*F*_(2,75)_ = 8.283, *p* = 0.0006) and not in the von Frey test (*F*_(2,73)_ = 2.001, *p* = 0.1426).

### The influence of metamizole on the levels of C1q/IBA1 and GFAP in DRG 7 days after CCI

According to RT-qPCR analysis, the *C1q* level was upregulated from 1 ± 0.1 to 4.2 ± 0.9 (*F*_(2,19)_ = 7.635; *p* = 0.0037) in rats subjected to CCI compared with naive rats (Fig. 2a). No significant differences in the *C1q* mRNA level were observed between the metamizole- and vehicle-treated rats after CCI (Fig. 2a). The mRNA level of *GFAP* in DRG was upregulated from 1 ± 0.1 to 1.7 ± 0.1 (*F*_(2,25)_ = 17.88; *p* < 0.0001) in vehicle-treated rats exposed to CCI to 1.88 ± 0.15 in naive rats and remained unchanged after metamizole administration (Fig. 2b).

Through Western blot analysis, we observed an increase in the IBA-1 protein level from 1 ± 0.07 in naive animals to 1.6 ± 0.1 (*F*_(2,19)_ = 7.032; *p* = 0.0052) in vehicle-treated rats after CCI (Fig. 2c). Repeated administration of metamizole in rats exposed to CCI slightly changed the IBA-1 protein level, but we did not observe significant differences between the vehicle-treated and metamizole-treated rats after CCI (Fig. 2c). The GFAP protein level was significantly higher in the vehicle-treated rats exposed to CCI than in the naive rats (1.5 ± 0.1 vs. 1 ± 0.1; *F*_(2,20)_ = 10.99; *p* = 0.0006). However, the GFAP protein level was unchanged by metamizole treatment (Fig. 2d).

### The influence of metamizole on IL-1beta, IL-18 and IL-6 levels in DRG 7 days after CCI

The mRNA level of *IL*-*1beta* was upregulated from 1 ± 0.06 to 1.7 ± 0.1 in rats subjected to CCI compared with naive rats (Fig. 3a). Metamizole significantly decreased the CCI-induced elevation in *IL*-*1beta* level from 1.7 ± 0.1 to 1.2 ± 0.1 (*F*_(2,25)_ = 14.97; *p* < 0.0001). Moreover, we observed significant upregulation of the *IL*-*18* level from 1 ± 0.03 to 1.52 ± 0.09 in rats after CCI compared with naive rats, and metamizole effectively diminished the IL-18 level from 1.5 ± 0.1 to 1.3 ± 0.1 (*F*_(2,23)_ = 12.57; *p* = 0.0002) (Fig. 3b). Additionally, the *IL*-*6* mRNA level was strongly upregulated from 1 ± 0.1 to 74.6 ± 3.7 in rats exposed to CCI compared with naive rats (Fig. 3c). The level of *IL*-6 decreased from 74.6 ± 3.7 to 61.0 ± 4.7 (*F*_(2,24)_ = 135.8; *p* < 0.0001) in rats that received repeated metamizole treatment compared with vehicle-treated rats subjected to CCI (Fig. 3c).

Western blot analysis showed that the protein level of IL-1beta in rats after CCI was unchanged compared with that in naive rats, and metamizole treatment did not affect the IL-1beta protein levels (*F*_(2,18)_ = 2.015; *p* = 0.1623) (Fig. 3d). However, at the same time point, we observed a significant increase in the protein level of IL-18 from 1 ± 0.1 to 1.4 ± 0.1 (*F*_(2,16)_ = 4.786; *p* = 0.0235) in the vehicle-treated rats compared with the naive animals, and metamizole administration did not significantly change the IL-18 protein level (Fig. 3e). Furthermore, we observed an increase in the protein level of IL-6 in the vehicle-treated rats exposed to CCI compared with the control group from 1 ± 0.2 to 1.4 ± 0.1, and administration of metamizole significantly diminished the CCI-induced elevation in the IL-6 protein level from 1.4 ± 0.1 to 1.03 ± 0.06 (*F*_(2,15)_ = 6.335; *p* = 0.0101) (Fig. 3f).

### The influence of metamizole on the studied chemokine mRNA levels in DRG 7 days after CCI

RT-qPCR analysis of DRG showed a significant increase in the level of *CCL1* from 1 ± 0.2 to 1.7 ± 0.3 (Fig. 4a), *CCL2* from 1 ± 0.1 to 2.2 ± 0.1 (Fig. 4b), *CCL3* from 1 ± 0.05 to 1.3 ± 0.1 (Fig. 4c), *CCL4* from 1 ± 0.04 to 1.3 ± 0.1 (Fig. 4d), *CCL6* from 1 ± 0.1 to 1.5 ± 0.6 (Fig. 4e), *CCL7* from 1 ± 0.03 to 1.4 ± 0.1 (Fig. 4f), *CCL9* from 1 ± 0.1 to 2.2 ± 0.3 (Fig. 4g), *CCL17* from 1 ± 0.1 to 1.7 ± 0.7 (Fig. 4h), *CCL22* from 1 ± 0.1 to 1.9 ± 0.25 (Fig. 4i), and *XCL1* from 1 ± 0.1 to 7.9 ± 0.6 (Fig. 4j) in vehicle-treated rats exposed to CCI compared with naive animals.

Compared to vehicle treatment, repeated ip metamizole administration significantly attenuated the mRNA levels of *CCL2* from 2.2 ± 0.1 to 1.8 ± 0.1 (*F*_(2,22)_ = 51.05; *p* < 0.0001) (Fig. 4b), *CCL4* from 1.3 ± 0.1 to 1.05 ± 0.1 (*F*_(2,21)_ = 5.709; *p* = 0.0105) (Fig. 4d) and *CCL7* from 1.4 ± 0.1 to 1.2 ± 0.1 (*F*_(2,20)_ = 7.11; *p* = 0.0047) (Fig. 4f) in rats after CCI. Additionally, we observed that metamizole did not influence the mRNA levels of *CCL1* (Fig. 4a), *CCL6* (Fig. 4e) or *CCL9* (Fig. 4g), with these levels approaching the levels observed in the naive group. In addition, unlike those of the abovementioned chemokines, the elevated *CCL1*, *CCL3*, *CCL6*, *CCL9*, *CCL17*, *CCL22*, and *XCL1* levels induced by CCI were not diminished by metamizole treatment (Fig. 4a, c, e, g–j, respectively).

## Discussion

Our study showed that metamizole administration reduces tactile and thermal hypersensitivity in rats after CCI. Our animal model mimics the characteristic features of neuropathic pain in humans [24] and has been used in our previous experiments [7–11]. Our behavioral results are in agreement with research by Nassini et al. [31] and Ince et al. [28], who found that metamizole not only prevents pain induced by scalpel incision in the rat paw but also diminishes tactile hypersensitivity in inflammatory (formalin, carrageenan) and neuropathic (partial sciatic nerve ligation) pain models. Moreover, it has already been shown that metamizole reduces hypersensitivity in neuropathy induced by diabetes [32] and cancer [33]. Here, for the first time, we showed that metamizole influences several immunological pronociceptive factors in DRG. To investigate the molecular background of the observed analgesic effects of metamizole, we chose to further study pronociceptive factors, which undoubtedly play an important role in nociception at the DRG level [5, 7–11, 14].

Primary afferent sensory neurons are responsible for nociceptive transmission, and their cell bodies are located in DRG. Data from previous studies indicate that resident macrophages are normally present in DRG, alongside satellite glial cells, and though remote from the lesion site, they react to nerve injury by releasing cytokines [34]. In the first week after nerve injury, macrophages flow into DRG [34]. Currently, it is known that in addition to resident and recruited immune cells present throughout the neuraxis, cytokines are released from neuronal cells [35]. Thus, cytokines compose a communication network between immune and neuronal cells. Interleukins (IL-1beta, IL-18 and IL-6) are recognized as the first pronociceptive cytokines in neuropathy [35] and are known to be strongly activated upon neural damage in the CNS and PNS [35–39]. In our previous studies, we showed that minocycline administered ip has an analgesic effect in parallel with diminished immunological factors, such as IL-1beta and IL-18 in DRG [40, 41]. Furthermore, it is worth noting that Botulinum toxin A, which was found to directly affect neuronal transmission, also decreases IL-1beta and IL-18 levels in DRG [41]. Numerous experimental and clinical studies have suggested that cytokines are good targets for therapy. ACZ885, a monoclonal antibody that neutralizes IL-1beta, is known to reduce rheumatoid arthritis symptoms in mice and humans [42]. Moreover, administration of anti-IL-1beta antibody [43] and IL-18BP [44] reduced neuropathic pain in rodents. There is solid clinical evidence supporting IL-6 as a potential therapeutic target in pain states [38]. Anti-IL-6 neutralizing antibodies, such as tocilizumab, clazakizumab, olokizumab, and sarilumab, are already being used in the clinic [45–47]. For example, two separate prospective cohort studies have suggested some benefit of tocilizumab in patients with low back pain [46, 47]. For postoperative pain, it has been demonstrated in humans that pretreatment with pentoxifylline can diminish the opioid requirement in the early postcholecystectomy operative period, as well as TNF-α and IL-1beta serum levels [48].

In our previous studies, we demonstrated for the first time that repeated administration of metamizole strongly suppresses the CCI-induced activation of microglial but not astroglial cells [23]. In vitro studies performed in a human monocytic cell line showed that metamizole also has an inhibitory effect on monocyte activation and can potently suppress chemokine production [49]. Here, we observed strong activation of monocytes/macrophages and satellite cells in DRG 7 days after sciatic nerve injury, which is in agreement with our previous findings [40, 41, 44]. However, metamizole did not significantly suppress the changes in these levels. In our previous studies [23], we demonstrated that metamizole did not suppress astrocyte activation. Thus, we can assume that the mechanism underlying the action of metamizole does not involve GFAP-positive cells in the nervous system. However, we observed that metamizole reduced the CCI-induced elevations in the mRNA levels of the pronociceptive IL-1beta, IL-6 and IL-18 cytokines. IL-1beta is known to be strongly activated at the DRG level upon neural damage [50], and intrathecal IL-1beta administration induces hypersensitivity in rats [4, 36, 51]. Recently, little evidence has been presented concerning the effect of metamizole on IL-1beta in neuropathic pain. In 2018, we showed [23] that metamizole diminishes the spinal level of IL-1beta in a mouse model of neuropathic pain. Similarly, Zhang et al. [22] showed that intracerebroventricular injections of metamizole strongly reduce the upregulation of IL-1beta in a cerebral ischemia model. IL-18 is another cytokine belonging to the IL-1 superfamily and has pronociceptive properties during the development of neuropathic pain [44, 52]. It has been shown that IL-18 is upregulated in spinal microglia after peripheral nerve injury and that an anti-IL-18 antibody attenuates injury-induced mechanical hypersensitivity in rats [39]. In the current study, we observed that CCI induces upregulation of *IL*-*18* mRNA and protein levels in DRG, which is consistent with other studies [40, 44, 53]. Moreover, we provided the first evidence that metamizole significantly prevents the CCI-induced upregulation of *IL*-*18* mRNA in DRG and that this upregulation may be crucial for the analgesic properties of metamizole. It is well known that IL-6 plays a key role in neuropathic pain development [4, 54]. Here, we confirmed that the level of *IL*-*6* mRNA is strongly enhanced in rat DRG after CCI, and similar results have been published previously [40, 50]. It was previously reported that administration of IL-6 neutralizing antibodies diminishes pain behavior in neuropathic pain models [55]. We demonstrated that metamizole significantly reduces the mRNA and protein levels of IL-6 in DRG, which might be one of the reasons for its beneficial properties.

Chemokines are a family of small chemotactic molecules responsible for attracting circulating leukocytes, especially granulocytes, lymphocytes, and monocytes, to the site of injury. They are produced by immune cells and by neurons, microglia and astrocytes [56]. Many chemokines from the CC-, CXC- and XC subfamilies are involved in nociceptive transmission [7, 9–11, 56–58]. Previously, it was demonstrated that some chemokines are upregulated after peripheral nerve injury not only in the spinal cord but also in DRG [11]. Moreover, our recent studies have shown that the most pronounced upregulation in DRG at 7 days post-CCI is observed for chemokines in the CC- subfamily [7, 11]. Thus, CC chemokines seem to be an interesting target for pharmacological modulation. Here, we confirmed the upregulation of *CCL1*, *CCL2*, *CCL3*, *CCL4*, *CCL6*, *CCL7*, *CCL9*, *CCL17*, and *CCL22* mRNA levels in DRG 7 days after peripheral nerve injury in rats.

In 2013, it was reported that CCL1 expression is increased in DRG after partial sciatic nerve ligation in mice [59]; this finding is in agreement with the results we observed in rats. It has also been shown that CCL1 is a crucial factor in the pathogenesis of neuropathic pain induced by diabetes [58]. Here, we did not observe any changes in the level of CCL1 after repeated administration of metamizole.

CCL2 is a significant pronociceptive mediator. Recently, Piotrowska et al. [9] and Kwiatkowski et al. [8] reported that modulation of the CCL2/CCR2 pathway by a CCR2 antagonist reduced neuropathic pain development in rats. Intrathecal administration of CCL2 leads to both thermal and mechanical sensitization, whereas injection of anti-CCL2 neutralizing antibodies suppresses the development of hypersensitivity [60, 61]. We have already shown that metamizole reduces the spinal level of CCL2 in a mouse model of neuropathic pain [23]. Our current results provide new evidence that CCL2 is undoubtedly involved at the DRG level in the analgesic effects of metamizole in neuropathic pain.

The chemokines CCL3, CCL4, and CCL9 are members of the macrophage inflammatory protein-1 (MIP-1) family. It has been previously shown that CCL3 and CCL4 are produced by activated macrophages and Schwann cells in the injured nerve; thus, they have been postulated to be key participants in the development of pain at the site of injury [62, 63]. Recent data have shown that intrathecal injection of CCL3, CCL4, or CCL9 evokes strong pain-like behavior in naive mice [64]. Additionally, direct neutralization of CCL3 or CCL9 decreases pain-related behaviors in diabetes- [64] and paclitaxel-induced neuropathy [65]. Furthermore, intrathecal injections of the selective CCR5 antagonist maraviroc significantly decreased the levels of CCR5 pronociceptive ligands, including CCL3 and CCL4, and thus reduced neuropathic pain symptoms after CCI of the sciatic nerve [11]. The findings of our present study show that the analgesic effect of metamizole is strongly associated with a reduction in CCL4 expression in DRG.

The role of CCL6 in neuropathic pain still needs to be clarified. Recently, we observed significant time-dependent changes in the CCL6 mRNA level in DRG starting at day 7 and lasting until day 28 after CCI [53]. In our current study, we observed that metamizole diminishes the CCI-induced elevation in the CCL6 level. Thus, little is known about the role of this chemokine in neuropathy, and further studies are needed.

In painful neuropathy, upregulated CCL7 levels have also been observed in DRG [11, 66]. Moreover, intrathecal administration of CCL7 strongly induces mechanical and thermal hypersensitivity in naive mice, and administration of neutralizing antibodies attenuates neuropathic pain in mice after CCI [67]. The results confirmed that CCL7 is strongly involved in the development of hypersensitivity in neuropathy. We provide the first evidence that metamizole significantly prevents CCI-induced upregulation of CCL7 in DRG, which may have an important impact on the analgesic properties of metamizole.

Recent studies have demonstrated increased expression of CCL17 in patients suffering from diseases that produce pain symptoms, such as fibromyalgia [68], autoimmune encephalomyelitis, and multiple sclerosis [69]. Moreover, Lee et al. [70] showed that CCL17 is important for the development of collagenase-induced osteoarthritis and suggested that neutralization of CCL17 might bring therapeutic effects [70]. Additionally, it was previously shown that CCL22 is involved in the pathogenesis of autoimmune encephalomyelitis [69]. For the first time, we have shown that *CCL17* and *CCL22* mRNA are upregulated in DRG after sciatic nerve injury; however, the levels of these mRNAs remained unchanged after metamizole administration. Thus, these chemokines are likely not involved in the beneficial effects of metamizole, and therefore, the involvement of CCL17 and CCL22 in neuropathic pain remains unknown.

Interestingly, for the first time, we observed strong upregulation of chemokines from the XC subfamily, namely, XCL1, at the DRG level. Our previous studies [57] showed that spinal XCL1 plays an important role in diabetic neuropathic pain. Moreover, we have previously shown that metamizole diminishes XCL1 expression at the spinal cord level in a model of neuropathy [23]; however, our current results indicate that this is not a case at the DRG level.

## Conclusions

Millions of people worldwide suffer from neuropathic pain as a consequence of nervous system dysfunction in the course of numerous diseases. Development of effective therapeutic strategies requires a better understanding of the molecular mechanisms underlying the pathogenesis of neuropathy. This study is an original step toward understanding the actions of metamizole in neuropathic pain. Our findings in a rat model of neuropathic pain provide evidence that metamizole exhibits beneficial analgesic effects by modulating the complex neuro-immunological interactions mediated by interleukins and chemokines at the DRG level. Our results show for the first time that pronociceptive factors, such as IL-1beta, IL-6, IL-18, CCL2, CCL4, and CCL7, play an important role in the analgesic activity of metamizole. Additionally, we suggest that the beneficial effects of metamizole result from a significant reduction in not only the levels of the abovementioned factors but also those of other chemokines, which are only slightly decreased after treatment. Overall, in our opinion, metamizole should be added to the modest arsenal of effective remedies for neuropathic pain.
